# 

**Published:** 1802-07

**Authors:** 


### THE MEDICAL AND PHYSICAL JOURNAL;

CONDUCTED BY

T. BRADLEY, M.D.

R. BATTY, M.D.

AND

A. A. NOEHDEN, M.D.

VOL. VIII.

FROM JULY TO DECEMBER, 1802, INCLUSIVE.

S.G.O. LIBRARY

*LONDON:*

PRINTED FOR

R. PHILLIPS, NO. 71, ST. PAUL'S CHURCH YARD;

AND SOLD BY ALL BOOKSELLERS IN THE UNITED KINGDOM.

Surgeon General's Office LIBRARY 5714 Washington, D.C.

[ William Thorne, Printer, Red Lion Court, Fleet Street. ]

### To CORRESPONDENTS.

*In future we wish those Gentlemen who send us Communications with anonymous signatures, to indicate their real names and address, in a private and confidential note to the Editors. Many valuable papers have necessarily been laid aside, from the circumstance of the names of the Writers being unknown to the Editors; and in a work of authority like the Medical and Physical Journal, in which the Editors take upon themselves the responsibility of all facts, for which a responsible Author does not appear, it is obvious they ought to be made acquainted with the real names of the authors of all the papers which appear in their work.*

*Communications and Books are received from Messrs.* Mawman, Morgan, Edmonston, Ricards, Ring, Crowfoot, Fowler, Dr. Langslow, Thornton, A. R. S. G. F. T. N. G. Anti-quackery, *and* Anti-empiricus.

*Dr.* Davis, *of Bath, complains of injustice in Mr.* Cuff's *evidence, as detailed in the Report of the Committee on Dr.* Jenner'*s Petition, No.* 13, *of the Appendix to the Report.*

---

### ERRATA.

Vol. VII. Page 450, line 17, *for* viſcus *read* veins.
520 and 521, *for* calx *read* calc.
521, *for* adeps *read* adep.
521, *for* creta *read* cretæ.

---

THE LONDON MEDICAL REVIEW *having this Month been discontinued for want of due Encouragement, the Readers of that Work and the Public at large are informed, that henceforward all new Books, connected with Medicine and Natural Philosophy, will be regularly analyzed in the Medical and Physical Journal. This Work, by its punctual Analysis of all new Books as fast as they appear, will consequently recommend itself to the Readers of the London Medical Review, and be increased in Value to its own immediate Subscribers.*

